# Contribution of *GLC3A* locus to Primary Congenital Glaucoma in Pakistani population

**DOI:** 10.12669/pjms.306.5771

**Published:** 2014

**Authors:** Rasheeda Bashir, Mahrukh Sanai, Adnan Azeem, Imran Altaf, Faiza Saleem, Sadaf Naz

**Affiliations:** 1 Rasheeda Bashir, PhD in Microbiology & Molecular Genetics, Assistant Professor, Department of Biotechnology, Lahore College for Women University, Lahore, Pakistan.; 2 Mahrukh Sanai, Student (MS in Biotechnology), University of Veterinary and Animal Sciences, Lahore, Pakistan. Department of Biotechnology, Lahore College for Women University, Lahore, Pakistan.; 3 Adnan Azeem, FCPS in Ophthalmology, Senior Registrar, Eye Dept at Mayo Hospital, Ophthalmology Department, Mayo hospital, Hospital Road, Lahore, Pakistan. Department of Biotechnology, Lahore College for Women University, Lahore, Pakistan.; 4 Imran Altaf, PhD in Microbiology, Assistant Professor, WTO, QOL, University of Veterinary and Animal Sciences, Lahore, Pakistan. Department of Biotechnology, Lahore College for Women University, Lahore, Pakistan.; 5 Faiza Saleem, PhD in Biology, Assistant Professor; 6 Sadaf Naz, PhD in Molecular Biology, Associate Professor, School of Biological Sciences, University of the Punjab, Quaid-i-Azam Campus, Lahore 54590, Pakistan. Department of Biotechnology, Lahore College for Women University, Lahore, Pakistan.

**Keywords:** GLC3A, Primary congenital glaucoma, Genetic linkage, Buphthalmos, * CYP1B1*

## Abstract

***Objectives:*** To check the contribution of *GLC3A* locus to primary congenital glaucoma in the Pakistani population.

***Methods:*** We enrolled twenty-nine sporadic cases and three families with multiple individuals affected with recessive primary congenital glaucoma in the year 2013. It was a genetic linkage study accomplished jointly in Department of Biotechnology of Lahore College for Women University and School of Biological Sciences, University of the Punjab, Lahore. Samples from all affected individuals were checked for homozygosity for alleles of microsatellite markers spanning *CYP1B1* at *GLC3A* locus. Genotyping was performed with fluorescently labeled primers by capillary electrophoresis. For familial cases, linkage was evaluated by checking the co-segregation of the phenotype with the genotypes. Two-point LOD score was calculated for each microsatellite marker with MLINK.

***Results:*** Our study revealed that *GLCA3 *may contribute to glaucoma in 17% of the sporadic cases and patients in 2 of the 3 families.

***Conclusions:*** This data suggests that the *GLC3A* may make an important contribution to autosomal recessive primary congenital glaucoma in the Pakistani population. Genotyping and Sequencing of more families will be helpful to identify the common mutations in *CYP1B1* in future.

## INTRODUCTION

Glaucoma is the second most common global disorder and the third important cause of blindness worldwide.^1^ According to National Health Survey of 2003, the incidence of blindness in Pakistan is 2.7%. Glaucoma is the fourth most common cause for reported blindness in Pakistan.^1^ British Infantile and Childhood Glaucoma study has shown that the incidence of Primary Congenital Glaucoma (PCG) in the Pakistani children is about nine times higher than that in Caucasians.^2^

Primary Congenital Glaucoma is an ocular disorder of early childhood. It is characterized by high intra-ocular pressure (IOP), corneal edema, photophobia, extreme tearing and enlargement of eye ball (buphthalmos).^3^ Primary congenital glaucoma mostly segregates as an autosomal recessive disorder.^4^

Up till now four loci have been mapped (*GLC3A, GLC3B, GLC3C, GLC3D*) and two genes (*CYP1B1, LTBP2*) have been identified for primary congenital glaucoma.^5^^,^^6^ Samples from patients in families with linkage to *GLC3A* have mutations in *CYP1B1.*^7^^,^^8^ In highly inbred populations like Slovakian Gypsies, Iranians and Saudi Arabians; 80-100% prevalence of recessively inherited glaucoma is reported to be due to mutations in *CYP1B1.* In three Pakistani families three novel and one previously reported mutations have been identified in* CYP1B1*. There is very little data available about the role of *CYP1B1* gene in Pakistani population. Therefore, the present study was designed to check the involvement of *GLC3A* locus to recessively inherited PCG in Pakistan.

## METHODS

Institutional review board (IRB) approval was obtained at Department of Biotechnology, Lahore College for Women University and School of Biological Sciences, University of the Punjab, Lahore. Informed consent was taken from all participants and parents in case of young children. Twenty-nine single affected individuals and three families (PKGM1, PKGM2 and PKGM3) segregating recessively inherited primary congenital glaucoma were recruited from different areas of Punjab in the year 2013. Detailed medical history was obtained from all individuals. Clinical assessment of primary congenital glaucoma was performed by Slit lamp, Tonometry, Gonioscopy, Perimetry and Retinal Nerve Fiber Layer Assessment (RNFL) at Mayo Hospital Lahore. It was a genetic linkage study which was jointly conducted at Department of Biotechnology, Lahore College for Women University Lahore and School of Biological Sciences, University of the Punjab, Pakistan.

DNA was extracted from blood samples using a non-organic method.^9^ Microsatellite markers’ positions were obtained from the Marshfield linkage map. PCR for microsatellite markers (*D2S2238* and *D2S1346*) were performed with 50 ng DNA in 10 µl volume by using M13-tailed primers for the markers and a FAM labeled M13 universal primer. The PCR reaction mixtures contained 1X buffer, 0.24 pmole of each primer, 2 mM MgCl_2_, 200 µM of each dNTPs and 0.15 units of Taq DNA polymerase (Thermo Scientific, Germany). The amplification was performed in MyCycler^TM ^Thermal Cycler (Bio-Rad, USA) Using a touchdown PCR protocol. The first PCR cycle had an annealing temperature elevated by 10^°^C from the calculated melting temperature of the primers. The annealing temperature was decreased by 1^°^C in each subsequent cycle from 65^°^C to 55^°^C in the following 10 cycles. The last 25 cycles were carried out at an annealing temperature of 55^°^C. Genotyping was carried out on an ABI PRISM 310 genetic analyzer. The analysis of data was carried out after electrophoresis using Gene Mapper v3.2 (ABI). The alleles were called as homozygous or heterozygous. For familial cases, linkage was confirmed by checking the co-segregation of the marker alleles with the disease phenotype and the haplotypes were constructed for each family demonstrating linkage by identifying the ancestral chromosomes. The MLINK program of FASTLINK package^10^ was used to calculate two-point LOD score for each of the markers using equal allele frequencies. The disorder was coded as fully penetrant with a disease allele frequency of 0.001. The LOD score (Z) was calculated at recombination fraction of *θ* = 0.

## RESULTS

Genetic linkage analysis revealed that the disorder in two out of three families was linked to *GLC3A*. Among the sporadic cases, five of the twenty-nine patients were homozygous for alleles of both markers at this locus. 

**Fig.1:**
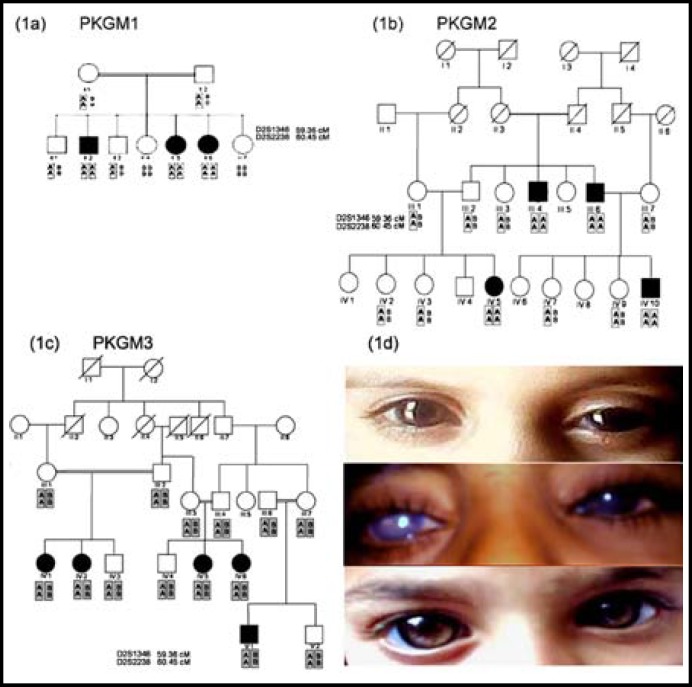
Families PKGM1, PKGM2 and PKGM3 with data of genotyping and images of patients’ eyes.

**a)** Pedigree of PKGM1 with haplotypes for markers on chromosome 2p21. Alleles of both markers were homozygous for affected individuals II:2, II: 5 and II 6, while obligate carrier were heterozygous.

**b)** Pedigree of family PKGM2 with haplotype data at chromosome 2 showing putative linkage of phenotype to* GLC3A* locus.

**c)** Pedigree PKGM3. The phenotype was not linked to* GLCA3* since the markers’ alleles were heterozygous. Solid filled squares and circles represent affected males and females respectively. The marker distances are in centimorgans (cM), arranged according to the Marshfield genetic map.

**d)** Phenotypes of eyes of affected individuals of families PKGM1 showed buphthalmos, corneal edema in both eyes while family PKGM2 patient had additional feature of photophobia. However, family PKGM3 showed clinical features of congenital glaucoma but a less severe phenotype was observed.

Family PKGM1 is a small nuclear family and was enrolled from Lahore. There are three affected individuals in this family (Fig.1a). The clinical features of congenital glaucoma which are buphthalmos, corneal edema and corneal scarring were present in all affected individuals of the family. The youngest patient II: 2 was 3 years old and had buphthalmos in both eyes with IOP of 7mm Hg /7mm Hg. Patient II: 5 had an IOP of 8 mmHg and 10 mmHg for right and left eye respectively. The visual acuity of patient (II: 5) was reduced to hand motion and no perception of light in the right and left eye correspondingly. Surgery was not performed on this patient and antiglaucoma medications were prescribed for him. Patient (II: 6) was a 10 years old child and had an advanced stage of congenital glaucoma. She also had buphthalmos, corneal edema and corneal scarring in left eye while her right eye had shrunken in size than normal eye. The visual acuity was reduced to hand motion and no perception of light. She had a high IOP of 32 mmHg and 30 mmHg for right and left eye respectively. Genotyping data of family PKGM1 showed all affected individuals were homozygous while unaffected individuals and their parents were heterozygous for alleles of markers *D2S2238 *and *D2S1346*. A maximum two-point LOD score of 1.6 at a recombination fraction of *θ* = 0 was calculated for marker *D2S2238*. Though the LOD score was less than 3, it was still suggestive of linkage as it was positive and the value was as expected for a small sized nuclear family 

Family PGKM2 (Fig.2b) was enrolled from Jhang and consists of four affected individuals. Individuals III: 4, III: 6 father and uncle of child IV: 10 were affected from glaucoma since birth but remained undiagnosed till now and therefore did not receive proper treatment. Patient IV: 10 was one years old and his IOP was 12/12 mmHg. The child had bilateral buphthalmos. Patient (IV: 5) is another affected child in this family and had 6 mm Hg/12 mm Hg IOP for the right and left eye respectively. The patient had corneal edema and photophobia. She had undergone augmented trabeculectomy and needed examination under anesthesia. These patients were prescribed antiglaucoma medications after surgery for control of glaucoma.

Genotyping was performed for all affected and available unaffected family members. All affected members were found to be homozygous for alleles of markers* D2S2238 *and *D2S1346*. The phenotypically normal individuals and parents were heterozygous. A maximum two-point LOD score of 1.8 at a recombination fraction of *θ* =0 was obtained for the marker *D2S2238* for this family. Though the family was large enough to support a high LOD score, the low score obtained may be explained due to the presence of the affected individual III:6 who is homozygous for alleles of both markers which consequently reduces power of detecting linkage in the subsequent generation. 

Family PKGM3 is a large inbred family enrolled from Sheikhupura (Fig.1d). This family had five affected individuals in three consanguineous unions. The symptoms of primary congenital glaucoma were noticed in all individuals at birth. These patients displayed the typical clinical features of congenital glaucoma which include corneal edema, excessive tearing, buphthalmos and photophobia. All affected and available unaffected members of the family were found to be heterozygous for the same alleles of markers* D2S2238* and *D2S1346* which therefore excluded linkage to *GLC3A*.

The twenty-nine sporadic cases with PCG, aged 1-3 years were enrolled from different area of the Punjab. Clinical assessment showed symptoms of high IOP ranges from 16 mmHg to 30 mmHg (mostly ≥21 mmHg), megalocornea, corneal edema, ruptures in the Descemet’s membrane and glaucomatous damage causing an alteration in the optic nerve head. A total of 5 individuals out of 29 patients were found to be homozygous for the alleles of both markers spanning *CYP1B1* at *GLC3A* locus which could be indicative of its involvement in the phenotype in this cohort.

## DISCUSSION

This is an initial study on the genetics of recessively inherited primary congenital glaucoma in Pakistan. Results of the study suggest that involvement of *GLC3A* is high in the Pakistani population and could be attributable to mutations in *CYP1B1*.

*CYP1B1* is the major contributor to primary congenital glaucoma in various ethnic groups.^11^^-^^13^ It is highly associated in inbred populations which includes, Slovakian Gypsies^14^ Turkish^15^ and Saudi Arabians.^16^^,^^17^ Previously, mutations in *CYP1B1* were identified in three out of thirteen or 23% Pakistani consanguineous families. able.

**Table-I:** Prevalence of congenital glaucoma in relation to GLC3A locus within different populations of the world

**Locus**	**Populations**	**Contribution to GLC3A locus (** ***CYP1B1*** **)**	**References**
*GLC3A*	Gypsy	100%	Plasilova et al.^14^
	Indonesia	33%	Sitorus et al.^7^
	Saudi Arabia	80%-100%	Bejjani et al.^16^; Abu-Amero et al.^17^
	Iran	70%	Suri et al.^12^
	Brazil	50%	Stoilov et al.^11^
	India	23%	Chakrabarti et al.^13^
	Pakistan	23%	Firasat et al.^3^
	Pakistan	2/3 Familial cases 17% Sporadic cases	This report

Our study demonstrates involvement of *GLC3A* locus (*CYP1B1*) in the sporadic individuals with PCG and about 17% of these subjects were found to be homozygous for alleles of markers linked to this locus. The number of families with PCG who participated in this work were too few to calculate the proportion of the phenotype due to mutations in *CYP1B1* but since the phenotype in 2 of the 3 families was consistent with linkage to *GLCA3*, it can be inferred that the contribution of this locus is quite high in this cohort, unlike that reported previously. However, it is possible that the actual contribution of *GLC3A* to familial PCG may be significantly lower or higher when large subsets of families are screened for mutations in *CYP1B1*. Screening additional familial cases with PCG will reveal the actual contribution of *GLC3A* locus and *CYP1B1* mutations to genetics of this disease.

In sporadic cases, frequency of homozygous samples to *GLC3A* locus was found to be 17%. It is possible that PCG of some sporadic individuals with the phenotype may not be genetic. We did try to minimize this possibility by completing a clinical history and only selecting affected individuals who were from consanguineous unions. Another possibility remains that some individuals could have compound heterozygous mutations in *GLC3A*. Nevertheless, the difference of primary congenital glaucoma in the two populations, one with clearly defined recessive mode of inheritance and the other in which recessively inherited deafness was inferred, supports the former possibility, rather than suggesting greater heterogeneity in sporadic cases as compared to the familial cases. Continued screening of the sporadic individuals for other PCG causing genes may clarify this issue in future. Direct DNA sequencing of the gene will also be helpful for detection and screening of *CYP1B1* mutations in Pakistani population which may be helpful in early detection and intervention of this disease. 

## Notes

***Source of funding:*** Higher Education Commission.
